# Designing new energy markets to promote renewables

**DOI:** 10.1016/j.heliyon.2024.e31427

**Published:** 2024-05-16

**Authors:** Giacomo Di Foggia, Massimo Beccarello

**Affiliations:** Department of Business and Law, University of Milano-Bicocca, Milan, Italy

**Keywords:** Market design, Distributed energy resources, Renewable energy sources, Green deal, Decarbonization, Energy policy

## Abstract

The drive toward decarbonization has spurred the growth of renewable energy sources, reshaping energy production and consumption patterns. As the energy landscape evolves, so must the market design supporting it to steer the integration of renewable energy. Addressing the challenges of promoting distributed renewable energy is paramount for developing a cleaner energy system and meeting decarbonization targets. This study presents a modern market design that efficiently integrates renewable energy sources, long-term contracts, and flexibility technologies into a single evolved market framework. The approach described herein provides proper price signals for diverse assets and decouples renewable energy from fossil fuels, ensuring economic viability and efficient integration. Taking into consideration key barriers and drivers, the findings provide insights for perfecting energy markets, meeting decarbonization targets, and guiding policymaking to boost cleaner energy systems.

## 1 Introduction

The rapid growth of renewable energy sources (RES) necessitates a significant structural overhaul of the design of the energy market [1]. Several pressing factors underscore the urgency of this transformation. Among these, the global commitment to decarbonize the energy system is foremost [2]—a need that is intensified by recent geopolitical shifts and energy market dynamics. Moreover, the increasing commitment to replace conventional thermal generation with RES reinforces the green transition [3], which is expected to yield positive economic outcomes [4]. However, the current energy market system faces challenges in integrating RES and optimizing economic resources and competition to ensure market sustainability.

Over the last two decades, the basis for the development of the European electricity market has been the so-called European Union (EU) target model for electricity markets, which is part of the EU's Third Energy Package aimed at minimizing barriers to competition and trade in the energy market [5]. However, the target model was designed to create a unified energy market with the objective of reducing the differences in energy price between member states—therefore, it originated in a different economic context, where the primary objective was the economic optimization of using different energy sources and generation technologies [6]. Since then, clean energy technologies have advanced rapidly [7] along with decarbonization targets and global commitment.

The energy mix is increasingly characterized by intermittent RES and flexibility technologies [8], i.e., solutions that enhance the adaptability of the energy system to varying conditions, with distinct operating models and cost structures necessitating a market design that is conducive to their growth and integration. In this context, the central research question of this paper is as follows: How can innovative market design effectively facilitate and enhance the integration of RES while simultaneously ensuring the stability and efficiency of the energy market, in alignment with decarbonization targets? This study proposes a framework comprising two key innovations to support this evolution. First, the framework promotes the negotiation of long-term contracts between renewable energy suppliers and consumers, reflecting the true costs of renewable generation. Second, it advocates the establishment of distinct markets for RES and flexibility technologies, thereby providing precise price signals for various energy resources.

Given the vulnerability of electricity markets to fluctuations in natural gas prices [9], it is crucial to decouple the RES market from the gas market to ensure efficient integration and economic viability. This urgency underscores the need to rethink market design in order to adapt to an evolving electricity system propelled by the transition to more environmentally friendly energy sources/green transition. The proposed framework, characterized by intermittent renewable generation and technologies offering flexibility, moves beyond the traditional approach of equating RES with conventional thermal generation.

In a RES-based system, there is a new structural need to ensure that the energy transition occurs efficiently: the planning of transmission and distribution networks must be closely linked to RES capacity [10] because transmission investment is a key policy instrument for facilitating this transition [11]. Although there is a substantial body of research on integrating RES, the specific mechanisms and market designs that can effectively harness their full potential remain underexplored. This study provides novel insights into renewable energy market design by delving into the integration of renewable sources and proposing specific market mechanisms and designs that exploit their full potential. By introducing a comprehensive renewable energy market structure, a tailored long-term contract platform, and a dedicated flexibility market, this study provides innovative solutions to the challenges of decarbonization and market integration. The findings thus offer a novel approach to energy market design, providing valuable insights for policymakers and industry stakeholders committed to a sustainable and efficient energy future in line with decarbonization targets.

Overall, decarbonization of energy generation and transformation of energy markets can yield several positive outcomes for society. Environmental benefits include reduction in greenhouse gas emissions and preservation of natural resources. Social benefits include improved public health, reduced dependence on imported fossil fuels, and increased community cohesion. Technological benefits include innovation in clean technologies, smart grid development, and the decentralization of energy production. Meanwhile, economic benefits include job creation, reduced electricity prices, and increased investment in clean technologies and infrastructure, all of which promote sustainable economic development.

This paper is structured as follows. Section 2 provides a review of the relevant background literature. Section 3 outlines the key elements of the proposed market design. Section 4 explores the potential evolution of the market. Section 5 presents a detailed discussion of the findings. The paper concludes with a summary of the key insights and implications.

## 2 Background

Globally agreed decarbonization targets aimed at limiting the increase in global temperature to 1.5° will require ambitious decarbonization policies [12,13]. Modernizing energy systems to steer the penetration of cleaner technologies necessitates both technological and organizational innovation [14]. A combination of factors and synergies between technological development, policy exertion, and societal attitudes [15] are becoming more urgent to achieve these targets. In this context, incentives, institutional change, removal of barriers, and engagement of key actors are deemed necessary to boost the decarbonization of energy generation and create modern markets [16].

Current market systems face several challenges in integrating RES into the energy market and governance. The integration of RES raises questions about optimizing the operation and governance of the energy system [17]. Developments in cleaner technologies have disrupted this discussion— with significant consequences for the nature of electricity markets, policy, and planning [18]—suggesting that new or additional market designs are required to ensure system adequacy in future power systems [19]. Estimating the impact of RES on the energy market is complex and the subject of ongoing debate—regardless of the market model used, rapid market changes make investment decisions challenging [20].

For example, the EU has committed to achieving ambitious goals by 2030 and 2050, aimed at the long-term mitigation of climate change [21]. The liberalization of European electricity markets began in the nineties, with one of the main objectives being to increase the efficiency of the electricity and gas supply by introducing competitive forces and regulations to integrate them into a single market. However, a single European electricity market has not yet been achieved because of structural and political barriers [22]. Notably, designing policies for electricity markets is complex, with market coupling being no exception [23]. As stated above, the development of a single market is currently underway. However, divergent national market designs pose a threat to the continuation of this process, even though previous literature confirms positive externalities to collectivity [24] and reports several potential benefits of market integration, including the efficiency of trading day-ahead, intraday, and balancing services across borders [25]. Given that the market harmonization and integration steps have been completed thus far [26], we argue that the development of a single European electricity market is set to intensify following the recent geopolitical energy crisis that has prompted the EU to accelerate the decarbonization path.

Decoupling RES from conventional thermal generation, especially from natural gas, has become urgent from a price formation perspective. Following the first surge in energy commodity prices due to post-pandemic economic recovery, several countries have experienced significant price increases despite having a relatively low share of gas in their energy mix [27]. Previous literature emphasizes that the dynamics of renewable generation are often tied to the prices of thermal generation, which are volatile over time [28]. This volatility can result in prices escalating in line with conventional sources' marginal costs [29]. However, the literature presents varied findings regarding the impact of thermal generation prices on renewable energy pricing dynamics. Notably, RES tends to be more expensive during the initial phases than conventional thermal generation [30].

To date, renewable and conventional energy generation technologies have coexisted within a single market. However, in a system dominated by conventional generation, plants are programmable and the value of electricity is mainly tied to generation costs. Conversely, in a mix with a significant renewable energy share, the value of electricity is influenced by the costs of managing challenges such as flexibility, variability, predictability, and controllability [31].

A step change in the transition to a more sustainable energy system must be facilitated through increased investment in clean energy generation [32]. It is necessary to develop a well-designed market model based on the inherent characteristics of the underlying generation system because integrating RES into a well-functioning electricity market can limit integration costs and boost investments in both generation and complementary flexibility technologies [33]. This would guarantee a balance between supply and demand, given that the more widespread the penetration of RES, the greater the volatility of supply [34]. Indeed, decarbonized energy systems are deemed feasible once flexibility requirements are met [35].

The role of prosumers and energy communities in shaping electricity markets is becoming prominent, and there is an increasing need to understand how they can be effectively and efficiently integrated into competitive electricity markets [36]. Indeed, the energy transition can catalyze greater involvement of individual consumers or citizens in community initiatives, based on local cooperation, that collectively act on the energy market [37]. Transmission system operator (TSO)-distribution system operators (DSO) coordination issues have recently gained notable ground among academic and practical interests [38]. Indeed, modern energy systems require optimally coordinated operation between transmission and distribution systems, given that, typically, the TSO solves its cost-optimization problem and evaluates the required targets for each DSO [39].

Such improved coordination is more important in light of the 2050 decarbonization targets [40]. Environmental objectives and the impact of increased energy prices have recently given rise to concerns about the competitiveness of European companies [41] under the current functioning of the market and price formation. In accordance with the additional future costs associated with decarbonization of energy production, the long-term additional costs for a higher share of renewable energy would be modest [42], as previously postulated by another study [43] that found an inverse relationship between RES penetration and electricity price. Given the different operating models of RES and flexibility technologies, a new market design is needed in which clean energy can be directly traded between producers and consumers with long-term contracts and prices that reflect the actual cost of generation. Energy and flexibility should be dealt with separately through markets that provide appropriate price signals for different technologies.

This study builds upon the previous literature by introducing an evolved market design that navigates the complex interplay between RES and flexibility technologies and aligns with the 2050 decarbonization goals. With its unique approach to addressing challenges and leveraging synergies between market actors, the proposed market design represents a significant leap forward in pursuing cleaner energy markets.

## 3 The framework

The proposed approach aims to achieve the desired future market design by rethinking markets to accommodate the role of decentralized RES. To establish a baseline, a case study—the Italian market design—was considered to contextualize how the proposed redefinition of the market may evolve. A multiple-case study approach was applied to obtain insights from successful initiatives on long-term contracts, flexibility, and auctions, including informed opinions of professionals to triangulate information [44] commonly used in energy and social research.

**Fig. 1:**
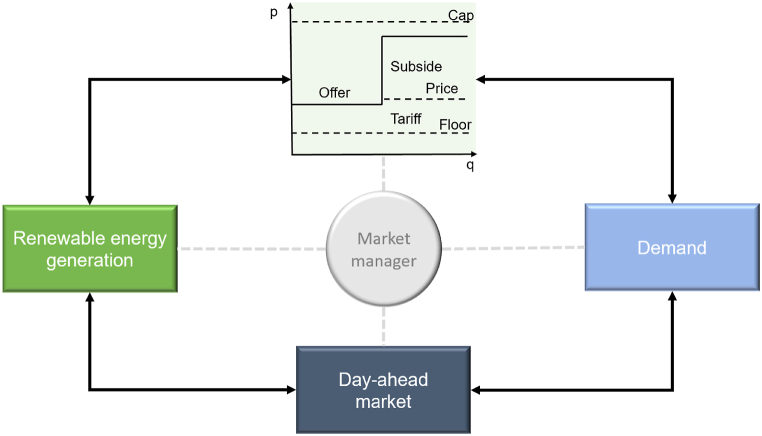
Renewable market operating scheme.

Source: Own elaboration based on [46,47].

The proposed framework comprises three pillars, the first of which is the renewable energy market. In the redesigned energy market, the paramount task is to guarantee adequate income for all generators while maintaining dispatch based on short-term marginal cost, considering the consequences of transitioning from a power system driven primarily by variable costs to one predominantly influenced by fixed costs [45]. This section introduces the renewable energy market, which is designed to promote RES and foster their integration by providing a dedicated platform for their valuation and trade. This approach seeks to benefit from the lower cost of renewable energy production, thereby leading to the decoupling of renewable energy from gas. The market is designed to facilitate participation through both public and private demand mechanisms. Indeed, a public counterpart manages such a market to ensure its operation and protect stakeholders. Counterparts trade actual volumes in the market, receiving and paying the respective prices in the spot market. Annually, contracted counterparts receive or pay the difference between their contracted price and the average spot price for their respective volumes. This mechanism applies to both sides of the market—i.e., those providing the energy and those consuming it—ensuring a balanced transaction with the market operator. Fig. 1 describes an approach to achieving a competitive market, while minimizing price volatility, by protecting producers and consumers through the use of a price cap.

The price cap regulates producer sales offers and consumer purchase requests, including maximum strike and floor prices. The strike price accounts for investment cost changes, the legalized cost of electricity for the involved technologies, and the producer's opportunity cost, also valuing the flexibility necessary to deliver renewable energy. Conversely, the floor price considers the cost of flexibility and ensures a minimum return for the producer. This market structure enables producers to obtain a fair return on investment while protecting consumers from excessive prices. The government encourages participation from traders, retailers, and independent renewable energy producers by providing a more stable environment. The central counterparts then underwrite financial contracts with demand and supply, awarded at the economic conditions offered, up to the volume at which equilibrium between supply and demand is achieved, as shown in Fig. 1. To address any gaps in achieving RES development targets, a system operator may launch subsequent extraordinary auction sessions based on the evolving liquidity of the renewable energy supply. These auctions promote participation by trading minimal volumes. Based on market conditions, their timing introduces functional information asymmetry, adding an element of risk for producers. The market design and principles are detailed in Table 1, which presents a summary of key auction mechanisms.

**Table 1:** Auction mechanism case studies.

Criteria	Portugal	Spain	UK	Netherland	Mexico	Brazil
Product type and volume	High	Moderate	High	Moderate	Medium	Medium
Payment	Medium	Medium	Medium	High	Medium	Medium
Counterparty risk	Medium	Medium	Medium	High	High	Moderate
Green technologies' contribution	Medium	Moderate	High	High	High	High
Level of competition	High	High	Medium	High	High	High
Impact on the system	Medium	Moderate	Moderate	Medium	High	High
Ease of implementation	Medium	High	High	Moderate	Moderate	Moderate
Model compatibility	Medium	Moderate	Moderate	High	High	High

Multiple case analyses support the design of some of the basic principles of renewable market operation such as procurement through long-term contracts, the presence of RES quotas on a zonal basis consistent with the development of the electricity system, the presence of locational price signals through zonal price differentiation, access to both supply and demand to allow contracting of energy directly between producers and consumers, and the presence of a central counterparty to manage counterparty risk.

**Fig. 2:**
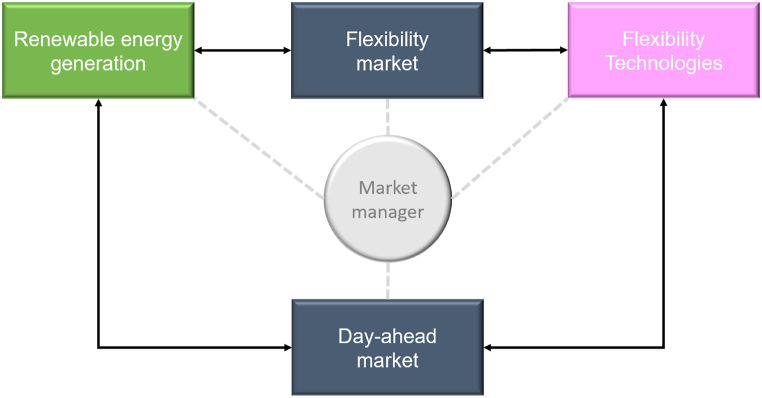
Example scheme of the flexibility market.

Source: Own elaboration based on [46,47].

The second pillar of the proposed framework is the flexibility market, which equips operators with the tools necessary to ensure the sustainability of products traded on the platform. The proposed market design offers diverse product types to satisfy various operators' risk-hedging needs and to provide price signals for the development of flexibility technologies. This is achieved as the gradual integration of flexibility assets takes place through participation in the flexibility markets themselves and in other spot and forward market segments offered by the new market design. The principle of technology neutrality supports an efficient market launch by first optimizing the use of existing resources and then gradually introducing more competitive and innovative technologies. Although current operator portfolios, including conventional thermal generation, provide some flexibility, the changing generation mix and the evolving costs of advanced technologies necessitate adjustments to meet the growing need for flexibility. Indeed, if the market design or incentives are not properly synchronized with existing renewable and storage capacities, storage could lead to higher system costs or emissions [50]. Assuming a simplified price settlement mechanism and annual settlement periods, the producer can effectively hedge its risk against a standard caseload product through time-swap products (as detailed in Fig. 2). Producers, as buyers of flexibility, provide compensation for these services and continue to trade in the day-ahead market. Finally, the producer sets a price for the contracted volume of the flexibility resource and trades a physical volume on the day-ahead market, securing a price differential.

The introduction of these mechanisms enables producers to manage price risk appropriately. If price settlement and adjustment periods occur annually, and time-swap products are captured, the producer fully hedges the product risk.

The third pillar is the long-term contract platform. The platform is poised to become the primary market for trading clean energy for new and existing capacities, where most of the products will be in the long term. This approach has the twofold effect of facilitating supply and demand matching and standardizing contracts. To mitigate counterpart risks, appropriate tools are introduced, including establishing a clearing house with a multilayered guarantee system that allocates credit risks to different parties, such as the operator and other clearing operators, and a special guarantee fund managed by the clearing house. The principles outlined in this paper, which underpin the development of fundamental operational principles for the platform, include securing procurement through long-term contracts, providing clear locational price signals, involving central counterparts to mitigate counterpart risk, and ensuring the transfer of benefits. The platform's supply liquidity will enable the negotiation of long-term contracts at prices aligned with the legalized cost of electricity derived from RES.

## 4 Development pathway

**Fig. 3:**
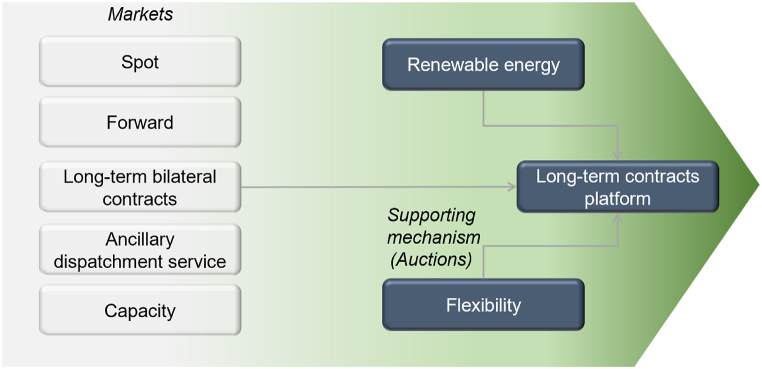
Expected development of the market design.

Source: Own elaboration based on [46,47].

To effectively arrive at the future market design, a gradual evolution from the current design is necessary. Fig. 3 depicts the structure of the markets and support mechanisms present to date and planned for the near future through current regulations. In the current European context, spot energy markets predominate in the market. In contrast, forward products, in exchange for long-term contracts, represent a minority share of the volume traded. Similarly, the dispatching services market and the spot balancing market are predominant in providing services to the grid. System adequacy needs are ensured through the capacity market, which offers long-term price signals to support existing plants and new investments. Finally, the supporting mechanisms—presented and projected—are in the form of auctions held around the development of generation plants and the large accumulation auctions provided for in EU Directive 944/2019. It is, however, worth noting that their complex design may raise concerns about biased effects against certain technologies [51]. The market setting described herein offers bankability conditions similar to those of traditional instruments, partly owing to the presence of central counterparts that allow the advantages of RES to be passed on to consumers through a price control system, thereby ensuring effective decoupling. Furthermore, this market design stimulates demand for flexibility and provides proper price signals while limiting—through the price control mechanism—the influence of commodities on the values of traded products.

The products traded in this market are designed based on medium-to long-term perspectives to enhance the bankability of investment projects. The market offers a diverse range of product types to cater to the varying risk-hedging needs of different operators and deliver precise price signals that encourage the development of green technologies, including hydroelectric storage and batteries. Within the ancillary services markets, the development of the forward dispatching services market ensures the system's security through long-term planning, while also offering market participants increased visibility into anticipated margins. The capacity market continues to be the designated instrument for ensuring system adequacy, addressing the need for both sufficient capacity as well as the requisite remuneration for this capacity. Once sufficient liquidity in the renewable and flexibility markets is achieved, the platform aims to facilitate energy trading over different time horizons and for any capacity.

### 4.1 Network management

To boost the development and integration of RES, there must be an evolution of the current operational management procedures for services in transmission and distribution networks, while preserving network security and cost-effectiveness for the system [52]. In other words, it is necessary to ensure that such development and integration take place at minimum cost and competitiveness in the markets, i.e., through limiting distortions and inefficiencies. To determine the best model for local resource management, certain priority characteristics are incorporated in the new design based on security, cost-effectiveness, and competition principles.

First, where beneficial to the system, the model must offer locally actionable services that can be operated in coordination with transmission network services. The provision of congestion resolution services on local networks may require the involvement of both TSOs and DSOs to optimize resolving local congestion issues while also considering the needs of the transmission grid. Second, implementation timelines should be compatible with decarbonization goals to effectively address grid security issues that may arise from the growth in RES and the increasingly active role of electricity demand. Third, the model should be reconciled with system operators’ investment plans in regard to networks, particularly investments suitable for network digitization.

Fourth, the model must ensure that the skills and tools of the system operator and the supply side meet the requirements of the management model. Finally, it should ensure the feasibility of data sharing and management of applied local services. In this context, in line with EU Directive 2019/944, a necessary condition for the DSO to play an active role in the management of network services in coordination with the TSO is the provision of adequate guarantees regarding decision-making autonomy, operational independence, and transparency of the activities performed. It is necessary to ensure that the system operator maintains neutrality regarding the selection and activation of distributed resources, in accordance with the principles of safety and cost-effectiveness for the system as well as its capacity to provide signals to traders and enable monitoring of the proper functioning of markets.

Optimal TSO-DSO coordination can be identified only by considering the system's safety, reliability, cost-effectiveness, and the competition between operators. The proposed scheme involves the creation of a local service market for the distribution network operated by DSOs alongside the existing market operated by the TSO. The local services market includes services such as congestion resolution and voltage regulation on low- and medium-voltage grids operated by DSOs. Distributed resources that offer such services include, among others, energy generation, batteries, electric vehicles, heat pumps, and energy communities. Several projects aimed at providing local flexibility services have already been initiated at the European level to evaluate the most appropriate and efficient solutions, with the involvement of both TSOs and DSOs. The European experiences analyzed in this paper mainly aimed to test the effectiveness and efficiency of services on distribution networks, especially congestion resolution services and voltage regulation on distribution networks.

### 4.2 Enabling factors

The transition to renewable energy has stimulated innovation in clean technologies and improved the efficiency, reliability, and affordability of renewable generation technologies over time. The integration of RES into grids has promoted the development of smart grids, which increase system flexibility, optimize energy use, and improve the overall reliability and resilience of energy systems. It is also worth noting that renewable energy and energy communities can promote the decentralization of energy production, thereby reducing the burden on centralized power plants and the grid. In addition, technological advances can help address the intermittency problem of renewable generation, further improving grid stability and reliability. The industry can create new jobs in manufacturing, installing, operating, and maintaining clean energy technologies. With the significant cost reductions in RES—particularly solar and wind resources—such clean technologies have become more affordable. Our proposed framework aims to accommodate the transfer of such benefits to citizens. If an ad hoc market is defined as expected in this paper, its benefits can be twofold: reducing consumer electricity prices and opening up investment opportunities in clean technologies and infrastructure, thereby stimulating economic growth and innovation.

The current market design limits distributed renewable energy production potential because of inappropriate price signals, lack of a dedicated marketplace, insufficient financial risk assurance, and unsuitable regulatory frameworks. Barriers include high upfront costs, lack of access to financing, regulatory hurdles, low public awareness, poor grid infrastructure, limited land availability, NIMBYism, and low conventional thermal generation prices. Solutions include providing grants, subsidies, and incentives; dedicated financing programs; streamlined permitting processes; public education campaigns; grid modernization; training programs; multifunctional land use; local stakeholder engagement; and a market design that differentiates renewable and conventional energy sources.

From Table 2, it is possible to conclude that decarbonizing energy production and transforming energy markets can yield several positive outcomes for society. The potential benefits of applying the proposed market design include the advantages that can be expected when the enabling factors, along with their corresponding drivers and barriers, are effectively managed and leveraged.

**Table 2:** Enabling factors and added value of the proposed framework.

Enabling factors	Barriers	Drivers	Proposal advantage
Upfront costs [[53], [54], [55]]	Distributed renewable energy projects often require significant initial investment, which can pose a barrier for smaller communities or those with limited financial resources.	Provide grants, subsidies, and tax incentives to reduce the initial financial burden and make projects more accessible to communities.	An ad hoc market providing medium-term price signals can encourage investment.
Access to financing [[56], [57], [58]]	Securing RES project funding can be challenging, particularly for smaller communities without established credit or solid financial backing.	Develop dedicated financing programs, such as green bonds or low-interest loans; provide more accessible funding options for smaller communities and projects.	Financial products are proposed in dedicated markets with high specialization levels.
Regulatory hurdles [[59], [60], [61], [62], [63]]	Complex regulations and permitting processes can hinder the development of renewable energy communities.	Simplify and streamline permitting processes and regulations for renewable energy projects, making it easier for communities to follow regulations.	Because renewable generation and flexibility markets differ structurally, specific regulatory frameworks are essential.
Public awareness [[64], [65], [66], [67]]	Poor understanding of the benefits of RES can make it difficult to garner public support.	Launch education and awareness campaigns to increase understanding of the benefits of RES.	Environmental output-driven communication is straightforward. Opportunities for high-value job creation
Grid infrastructure [[68], [69], [70]]	Many energy grids are not designed to accommodate RES, which can create challenges for integrating new development projects.	Implement grid modernization and smart grid technologies to facilitate the integration of RES and improve overall grid stability.	Flexibility and digitalization investment projects
Land availability [[71], [72], [73], [74]]	RES projects often require large amounts of land, which can be a challenge in densely populated areas with limited suitable land.	Encourage multifunctional land use to maximize land use efficiency and minimize conflicts.	Public administrators should integrate decarbonization targets into strategic urban development plans.
Nimby [[75], [76], [77], [78]]	Local opposition to renewable energy projects can be a significant barrier.	Engage local stakeholders and promoting the benefits of RES to foster community acceptance and support	Disseminating the benefits of scope and scale economies
Interaction with conventional thermal generation	The current market design links the renewable energy price to conventional thermal generation.	Implement a market design that considers the differences between renewable and conventional energy sources.	Effectively pricing the marginal cost of production by decoupling from natural gas
Complexity [79,80]	A common characteristic of these ecosystems is their complex composition, which often involves the interaction of multiple actors.	Provide a clear definition of roles, specifically regarding opt-in and opt-out procedures.	Clear definition of rules and limiting transaction costs
Network management [[81], [82], [83], [84]]	Coordination is difficult	Implement innovative business models; engage in data sharing and digitalization.	Facilitated interactions between DSOs and RES owners

## 5 Discussion

This study advances energy market design by discussing a comprehensive framework that more effectively and efficiently integrates RES into the market. Our findings challenge and build upon the literature on renewable energy development. This study has similarities with previous literature that provides policy recommendations for the market design of a future European electricity system [85]. Planning for the transmission and distribution grid of a RES-based system should closely be aligned closely with the growth rates of renewables to ensure an efficient transition [39]. Consequently, the market requires appropriate instruments to manage renewable generation, energy storage, and the creation of smart and resilient grids [81]. To achieve decarbonization goals, the structure of the energy market needs to be modified, and new structured markets must be defined and implemented [86]. To ensure cost-effectiveness, a rethinking of market design is necessary to align with the characteristics of the electricity system envisioned by the green ecological transition [17]. This involves reconciling the intermittent nature of RES with the flexibility required to adapt generation to demand.

The pathway to decarbonization entails profound structural changes in electricity generation technology, necessitating a major structural overhaul of the organizational structure of markets. However, the complexity of markets [80] and international differences have made this process slow and not without errors and inefficiencies. Recent events have significantly increased the urgency of this transformation, necessitating a step change to revise market rules in order to accommodate decarbonization.

Structural reform of the current market design is needed to ensure adequate progress toward achieving these goals, given that the current market design was developed with the aim of optimizing thermoelectric generation costs [87]. However, today, this approach proves ineffective in integrating renewable generation. The main challenge in the coming years will be the transition from old to new market rules, ensuring that decarbonization goals are achieved without technical or economic inefficiency [46].

Furthermore, this research contributes to the broader energy policy and economic development discourse, advocating for a more sustainable and secure energy future. The policy implications for decarbonizing energy production and redesigning energy markets derived from this study are as follows. First, we suggest that the current market design should be restructured to promote efficient integration and economic viability of RES. By decoupling renewable energy and flexibility markets, policymakers can ensure the proper integration of RES while maximizing the benefits of the transition. Second, the introduction of a long-term market for renewable energy can provide zonal price signals, stimulate the cost-effectiveness of technologies, and allow the benefits of lower marginal costs of renewable generation to be passed on to consumers. Third, policymakers should develop a trading platform that provides long-term price signals for renewable energy, considering the evolving costs of cleaner technologies. Fourth, a dedicated market for flexibility is needed to complement the platform and provide operators with the tools necessary to ensure the sustainability of the products traded on the platform. This market should offer diverse product types to meet the risk-hedging needs of different operators and provide appropriate price signals for developing and investing in flexibility technologies. Fifth, policymakers should ensure that existing dispatch service and capacity markets evolve to accommodate the emerging markets. In addition, transmission and DSO must closely coordinate their efforts to integrate renewable and flexibility technologies while ensuring network security and cost efficiency. In addition to providing important insights, this study opens up new avenues for future research, particularly in exploring empirical analysis based on decarbonization scenarios. By critically evaluating our findings within the broader field of energy market design, we offer a robust and forward-looking contribution to the ongoing evolution of energy markets.

Therefore, this research adds value by contributing an innovative approach to energy market design, with relevant insights for current European policy discussions. The proposed market design recognizes the structural differences between renewable generation and flexibility technologies, advocating a dichotomy of markets that reflects their different characteristics. This may foster more efficient integration of RES, while providing proper price signals. The practical implications of this framework may be significant given that long-term price signals and the development and competitiveness of renewable sources pave the way for an accelerated transition to a sustainable energy future. To this end, this study addresses well-known barriers to distributed renewable energy production by presenting solutions that consider RES, energy flexibility technologies, and ancillary services.

Overall, the proposed market design advances academic discourse by presenting a practical, forward-looking blueprint for reshaping energy markets in line with decarbonization goals [88] and the increasing demand for clean energy.

Although suggesting a forward-looking perspective, this study acknowledges several inherent limitations. Primarily, the geographical focus is on the European context; specifically, the starting point was the Italian case. The proposed framework may not directly translate to other regions with different regulatory environments, market dynamics, and energy infrastructures. As a conceptual framework, it is a prospective proposal rather than an evidence-based conclusion. The assumptions regarding market behavior, the integration of RES, and the effectiveness of the proposed markets are based on current understanding and projections, which may evolve with technological advancements and policy changes. While the framework is adaptable and scalable, its practical implementation requires testing, validation, and modification to address the dynamic nature of energy markets.

## 6 Conclusion

This study articulates a novel market design to discern the differences between renewable generation and flexibility technologies that are prominent in modern energy markets. It advocates for a market system that mirrors their distinct characteristics. By instituting this innovative market design, market operators can trade long-term contracts that reflect the true costs of RES while creating distinct markets for energy and flexibility. This facilitates the integration of RES and generates the right price signals. The proposed market design, which considers RES, flexibility technologies, and ancillary services, presents a comprehensive solution to the barriers that impede distributed renewable energy production. The following drivers and barriers to enabling factors are analyzed: upfront costs and access to financing, regulatory hurdles, public awareness, grid infrastructure and network management, land availability and NIMBYism, interaction with conventional thermal generation, and complexity. This study delineates a transformative approach to energy market design, underscoring the specialized structure and regulatory requisites of RES and flexibility markets. The present framework is based on decoupling RES from the volatility of conventional thermal generation. These features collectively accentuate the significance of this research, offering a forward-looking perspective for a decarbonized energy market.

## Data availability statement

No new data were generated or analyze, and any reference to data in this study are cited within the article.

## CRediT authorship contribution statement

**Giacomo Di Foggia:** Writing – review & editing, Writing – original draft, Methodology. **Massimo Beccarello:** Writing – review & editing, Writing – original draft, Resources, Methodology, Conceptualization.

## Declaration of competing interest

The authors declare that they have no known competing financial interests or personal relationships that could have appeared to influence the work reported in this paper.
